# Caring for the invisible and forgotten: a qualitative document analysis and experience-based co-design project to improve the care of families experiencing out-of-hospital cardiac arrest

**DOI:** 10.1007/s43678-023-00464-8

**Published:** 2023-02-13

**Authors:** Tess Loch, Ian R. Drennan, Jason E. Buick, Danielle Mercier, Peter G. Brindley, Mark MacKenzie, Thilo Kroll, Kate Frazer, Matthew J. Douma, Family Centred Cardiac Arrest Care Project, Samina Ali, Sheila Early, Kim Ruether, Kristin Flanary, Katherine E. Smith, Katie N. Dainty, Timothy A. D. Graham, Samir Z. A. Graham, Lynn Blomheart, Jesse Blommaert, Calah Myhre, Ella MacKenzie, Louise Jarratt, Katherine E. Smith

**Affiliations:** 1 University of Calgary, Cumming School of Medicine, AB Calgary, Canada; 2 Sunnybrook Centre for Prehospital Medicine, Sunnybrook Research Institute, Toronto, ON Canada; 3 Division of Emergency Medicine, Department of Family and Community Medicine, Temerty Faculty of Medicine, University of Toronto, Toronto, ON Canada; 4 Institute of Health Policy, Management and Evaluation, Dalla Lana School of Public Health, University of Toronto, Toronto, ON Canada; 5 Alberta Health Services, Edmonton, AB Canada; 6 Department Critical Care Medicine, Faculty of Medicine and Dentistry, University of Alberta, Edmonton, AB Canada; 7 School of Nursing, Midwifery and Health Systems, University College Dublin, Dublin, Ireland

**Keywords:** Cardiac arrest, Family-centred care, Emergency medical services, Qualitative, arrêt cardiaque, soins centré sur la famille, services médicaux d’urgence, qualitative

## Abstract

**Objectives:**

The objectives of this project were to collect and analyze clinical governance documents related to family-centred care and cardiac arrest care in Canadian EMS organizations; and to improve the family-centredness of out-of-hospital cardiac arrest care through experience-based co-design.

**Methods:**

We conducted qualitative document analysis of Canadian EMS clinical governance documents related to family-centred and cardiac arrest care, combining elements of content and thematic analysis methods. We then used experience-based co-design to develop a family-centred out-of-hospital cardiac arrest care policy and procedure template.

**Results:**

Thirty-five Canadian EMS organizations responded to our requests, representing service area coverage for 80% of the Canadian population. Twenty documents were obtained for review and six overarching themes were identified: addressing family in event of in-home death, importance of family, family member escort, provider discretion and family presence discouraged. Informed by our qualitative analysis we then co-designed a policy and procedure template was created that prioritizes patient care while promotes family-centredness.

**Conclusions:**

There were few directives to support family-centred care by Canadian EMS organizations. A family-centred out-of-hospital cardiac arrest care policy and procedure template was developed using experience-based co-design to assist EMS organizations improve the family-centredness of out-of-hospital cardiac arrest care.

**Supplementary Information:**

The online version contains supplementary material available at 10.1007/s43678-023-00464-8.

## Résumé

**Objectifs:**

Les objectifs de ce projet étaient de recueillir et d’analyser des documents de gouvernance clinique liés aux soins centrés sur la famille et aux soins de l’arrêt cardiaque dans les organisations canadiennes de SMU; et d’améliorer le caractère centré sur la famille des soins en cas d’arrêt cardiaque à l’extérieur de l’hôpital grâce à une co-conception fondée sur l’expérience.

**Méthodes:**

Nous avons effectué une analyse qualitative des documents de gouvernance clinique des SMU canadiens liés aux soins axés sur la famille et aux arrêts cardiaques, en combinant des éléments de contenu et des méthodes d’analyse thématique. Nous avons ensuite utilisé la co-conception fondée sur l’expérience pour élaborer un modèle de politique et de procédure de soins en cas d’arrêt cardiaque centrés sur la famille en dehors de l’hôpital.

**Résultats:**

Trente-cinq organisations Canadiennes de SMU ont répondu à nos demandes, ce qui représente une couverture de zone de service pour 80 % de la population canadienne. Vingt documents ont été obtenus aux fins d’examen et six thèmes principaux ont été cernés: s’adresser à la famille en cas de décès à domicile, l’importance de la famille, accompagnement d’un membre de la famille, la discrétion du fournisseur et la présence de la famille découragée. Éclairés par notre analyse qualitative, nous avons ensuite co-conçu un modèle de politique et de procédure qui priorise les soins aux patients tout en favorisant l’orientation familial.

**Conclusions:**

Il y avait peu de directives pour soutenir les soins axés sur la famille par les organisations canadiennes de SMU. Un modèle de politique de soins d’arrêt cardiaque centré sur la famille a été élaboré à l’aide d’une co-conception basée sur l’expérience pour aider les organisations de SMU à améliorer l’orientation familiale des soins en cas d’arrêt cardiaque hors hôpital.

## Clinician’s capsule

**Table:** 

***What is known about the topic?***
Cardiac arrest of a loved one has lasting impacts on families. When successfully adopted, family-centredness is associated with higher perceived quality of care
***What did this study ask?***
What clinical governance documents exist among Canadian EMS organizations with regards to family-centred cardiac arrest care?
***What did this study find?***
There are very few clinical documents to support family-centred cardiac arrest care in Canadian EMS organizations
***Why does this study matter to clinicians?***
This study uses qualitative document analysis and experience-based co-design to create a policy and procedure template that describes practical strategies to improve the family-centredness of out-of-hospital cardiac arrest care

“I was doing the compressions you know, the CPR like the person on the phone was telling me to do, but when the paramedics arrived they took me out of our bedroom, they wouldn’t let me be with him anymore, they kept me away from my own husband, in our bedroom, and I lost that time with him, I will never get it back.”—Family-centred cardiac arrest care project collaborator #17.

“The paramedics were amazing, they were so good, they explained to me what they were doing and why, they told me my Mum had like ventricular fibrillating and they shocked her. The supervisor, she even drove me to the hospital while the crew took Mum in the ambulance. I wish I could let the crew know how much I appreciated all they did for Mum and me”—Family-centred cardiac arrest care project collaborator #21.

Over 100,000 Canadians are bereaved by the sudden cardiac arrest of a family member every year [1]. Over the past 30 years there have been important advances in out-of-hospital cardiac arrest care with modest improvements in survival [2, 3] and little consideration of families. The long-term impacts of these events weigh heavily in the form of physiological and psychological stress [4, 5]. One year after a family member’s cardiac arrest, 40% of relatives experience after-effects such as disordered mood, post-traumatic stress, and anxiety [6, 7]. Mental health experts suggest the degree of symptomatology in family members may often be greater than the survivor of cardiac arrest [8].

Family-centred care is the collaboration of healthcare providers, patients, and families to optimize the quality of care [5]. It is well established in critical care [9] and paediatric emergency [10]. Family-centred care strategies in cardiac arrest care are believed by some to be a strategy to improve outcomes for patients and families [11, 12] yet barriers to their adoption in the prehospital setting have been identified [13].

A core component of a family-centred approach to cardiac arrest care is the facilitation of family presence [14]. However, offering presence during out-of-hospital cardiac arrest can be controversial as providers fear criticism or negative impacts on their performance [4, 13, 15]. The existence of family-centred cardiac arrest care policy, procedure or other clinical governance documents in Canadian EMS organizations is largely unknown. The overall aim of this project is to promote the adoption of family-centred cardiac arrest care into the prehospital setting.

The objectives of this project were to (i) collect and analyze clinical governance documents supporting family-centred care during out-of-hospital cardiac arrest in Canadian EMS organizations; and (ii) partner with survivors and family members to improve the family-centredness of out-of-hospital cardiac arrest care through the experience-based co-design of a policy and procedure template.

## Methods

### Study population and setting

We set out to obtain a representative sample of Canadian EMS organizations’ clinical governance documents (policies, procedures, protocols, guidelines etc.) We emailed Canadian EMS organizations asking for any documents related to family-centred care and cardiac arrest care. However, we accepted any documents that addressed family-centred care in general (presence, visitation, transport/escort) (see appendix for email template). We also conducted a Canadian grey literature search. We identified and emailed EMS organizations through the membership lists of the Canadian Resuscitation Outcomes Consortium (CanROC) and the Paramedic Chiefs of Canada. The province of Ontario required additional network sampling methods via the Ontario Association of Paramedic Chiefs website as they utilize over 50 individual services.

Collaborators with lived experience were engaged from the Family Centred Cardiac Arrest Care Project (https://osf.io/fxp5g/). This team has performed literature reviews and utilized experience-based co-design to developed various tools to improve cardiac arrest care [16, 17]. Twenty one persons with lived experience of cardiac arrest care from five Canadian provinces, (five survivors, eight family members of survivors and seven family members of non-survivors) participated in consultant, collaborator or co-investigator roles. They come from varied backgrounds, but include multiple practicing and retired healthcare professionals (physicians, nurses and allied health), a university professor, a theologian, a veterinarian, an unpaid caregiver, a pastor, a student, an artist and a yoga teacher.

### Study design

We utilized qualitative document analysis methods for this study, a health policy research methodology [18]. The systematic four-step “R.E.A.D.” procedure was used to guide our research: (1) (R)eading of clinical governance documents, (2) (E)xtracting of relevant data, (3) (A)nalyzing data and (4) (D)istilling any findings, using meta-synthesis [18]. We reported this study in accordance with the Qualitative the Guidance for Reporting Involvement of Patients and the Public (GRIPP2) and the Standards for Reporting Qualitative Research (SRQR) (see appendix for both reporting documents) [19, 20].

**Fig. 1:**
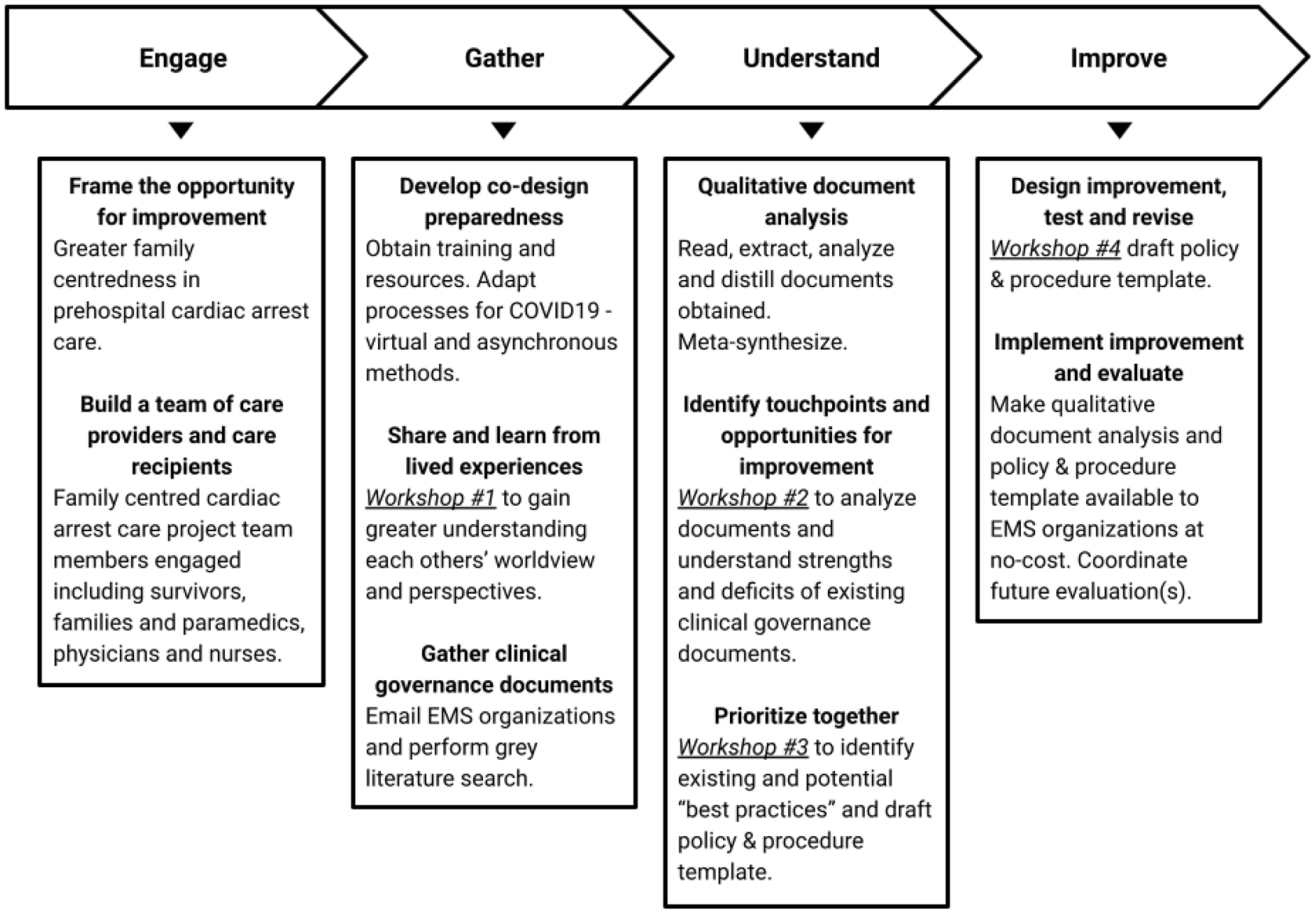
Experienced-based co-design process for family-centred cardiac arrest care improvement. This figure describes the experience-based co-design and qualitative document analysis process we undertook

Experience based co-design methods, (informed by our qualitative document analysis), were then used to develop a policy and procedure template. This type of co-design method is a form of participatory research utilizing lived experience of care providers and care recipients to improve care [21]. The method has also been described as a way for organizations to engage with care providers and care recipients to improve together [22]. Co-design workshops were undertaken during the COVID-19 pandemic using online synchronous and asynchronous tools such as Zoom [San Jose, California USA] and Jamboard, Draw, Docs and Sheets [The Alphabet company, Mountain View, California, USA]. Our co-design process had four phases: engage, gather, understand and improve [23]. See Fig. 1 for details.

### Data collection

Each identified EMS organization was emailed up to three times between April 2020 and April 2021 (see appendix for email template) and invited to contribute clinical governance documents (policy, procedure, protocol or guidelines etc.) related to family-centred care and cardiac arrest care (see appendix for email invitation). If three attempts resulted in no reply, they were marked as “no response”. Our grey literature search had three phases: geographically restricted internet search engine (anonymous browser for de-personalized search without individual user bias), grey literature database (16 Canadian sources) and targeted website searching [24, 25]. See appendix (tables five, six and seven) for search strategy.

### Data analysis

We conducted qualitative document analysis combining elements of content and thematic analysis [21]. Content analysis was used to identify, count and collate sections of document text. Thematic analysis was used to identify and examine patterns within and between the documents, developing key themes representative of the direction Canadian EMS care providers receive regarding family-centred and cardiac arrest care.

**Fig. 2:**
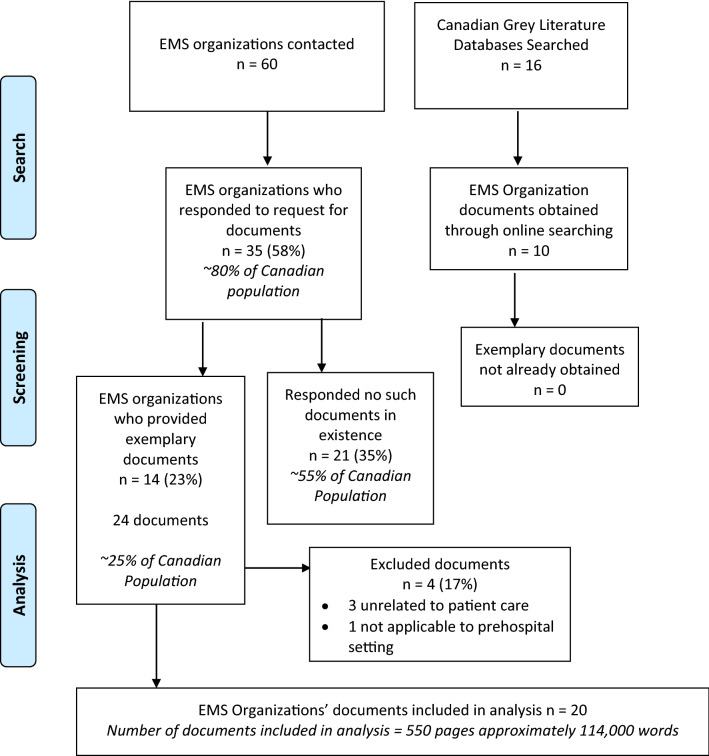
Document review flow diagram. This flow diagram describes our document search and screening

Documents were read repeatedly to develop in-depth knowledge of the data and searched to highlight key terms related to family-centred care (see table four appendix for key words used). Throughout the analysis phase, notes and codes were created and underwent further analysis until themes were identified and meta-synthesized. Meta-synthesis entailed grouping codes, comparing themes across documents, matching themes and generating higher-order analytical themes to go beyond the original documents and provide a superordinate interpretation [26]. Then, in partnership with survivors and family members with lived experience of out-of-hospital cardiac arrest, we performed co-design workshops, producing a universal policy and procedure template for family-centred cardiac arrest care. See Fig. 2 for a summary of our methods.

### Ethics

This project was exempt from full ethics review (Alberta Research Ethics Community Consensus Initiative Ethics Screening Tool https://arecci.albertainnovates.ca/ethics-screening-tool/, “Low Risk”) as there were no human research participants—our partners with lived experience were engaged as consultants, collaborators and co-investigators. Documents were collected from EMS organizations under the condition of anonymity.

## Results

### Qualitative document analysis

Sixty EMS organizations were contacted. Their “responses” were categorized as (i) no response (*n* = 25, 41.6%), (ii) positive response with clinical governance documents provided (*n* = 14, 23.3%) and (iii) negative response without clinical governance documents provided (*n* = 21, 35%). 35 (58%) of the EMS organizations responded to our request for clinical governance documents. These 35 services provide care for over 80% of the Canadian population, or approximately 30 million Canadians and include all provincial service providers (excluding Quebec). The entirety of the non-responses were from regional services within Ontario. No additional clinical governance documents were obtained through grey literature search. See Fig. 2 for flow diagram. In total, 20 clinical governance documents from 14 different services were obtained (see Table 1 for results summary). Four documents were excluded upon receipt because they irrelevant to family-centred care or cardiac arrest care.

**Table 1:** Description of Canadian EMS Organization documents obtained

EMS organization	Document type(s)	Details	Issued (revised)
Service 1	Guideline	21 pages, 6505 words	May 2018 (May 2020)
Service 2	Protocol	230 pages, 45,533 words	2020
	Protocol	91 pages, 18,402 words	2019
	Protocol	151 pages, 29,424 words	2020
Service 3	Policy	1 page, 305 words	August 2018
	Policy	1 page, 502 words	February 2018
Service 4	Policy	3 pages, 799 words	October 2018
	Guideline	1 page, 471 words	May 2020
Service 5	Policy	3 pages, 800 words	Sept 2009 (October 2018)
	Policy	8 pages, 1990 words	March 2013 (March 2020)
Service 6	Policy	6 pages, 971 words	January 2015 (January 2016)
Service 7	Policy and Procedure	6 pages, 1683 words	July 2003 (April 2019)
Service 8	Policy and Procedure	7 pages, 2023 words	No date
Service 9	Policy and procedure	3 pages, 835 words	January 2014 (April 2019)
	Policy and procedure	3 pages, 680 words	May 2010 (April 2019)
Service 10	Policy	2 pages, 494 words	June 2005
Service 11	Policy and procedure	2 pages, 514 words	January 2002 (June 2016)
Service 12	Procedure	1 page, 322 words	September 2006
Service 13	Policy	5 pages, 1030 words	December 2007
Service 14	Policy	4 pages, 943 words	November 2017
Summary data	Total number of documents = 20 Policy and/or Procedure = 15 (75%) Guidelines = 2 (10%) Protocols = 3 (20%)	Total pages = 549 Total words = 114,226	Date range (most recent revisions) = 2005–2020 Average year of publication = 2016

Five distinct and important themes were identified through our analysis: (1) Acknowledge the family when there is a death in the home; (2) Family members are important to the patient and patient care; (3) Family member escort options; (4) Provider discretion for family involvement; and (5) Family presence discouraged. Note, individual clinical governance documents could address more than one theme.

- 1. Acknowledging family in event of in-home death

Four clinical governance documents guided the social-emotional approach EMS providers were to employ for a death in the home, but not cases of active resuscitation. These documents advised EMS providers to be sensitive towards the family in these scenarios. However, there were no actionable steps or guidance to assist providers in navigating these situations.

- 2. Generalized importance of family

This theme included mentions of the term family-centred care, or addressed the patient and family dyad (for example, using terms like ‘patient and family’ rather than just patient). There were no specific actionable items, but an organizational culture that considered the patient and their family as a cohesive unit. Six documents provided guidance related to this theme and one was in the context of cardiac arrest care.

- 3. Family member escort option

Eight of the 20 clinical governance documents identified and supported circumstances when EMS providers could facilitate taking a family member in the ambulance with the patient. The content of these policies and protocols addressed the need for a seatbelt, adherence to traffic laws and required family member to not be intoxicated or aggressive.

- 4. Provider discretion

Six documents stated that the final decision to allow family presence was up to the EMS providers. In these examples, there was single statement explaining that the provider(s) had the discretion to determine the degree of closeness or involvement a family member could have while emphasizing that the EMS provider had the final say.

- 5. Family presence discouraged

In three cases, family presence was discouraged. In one example, family presence was discouraged outright. In the other two documents, family members or support persons were encouraged to find their own transport and limited patient accompaniment was emphasized in high priority calls.

See Table 2 for a complete description and example quotes that exemplify themes.

**Table 2:** Themes and exemplary quotes from Canadian EMS clinical governance documents

Theme	Number of services (percentage of services)	Service addressing theme	Exemplary quotes
1. Addressing family in event of in-home death	4 (28.6%)	1 2 7 9	“Review the steps for family/next of kin to take at the time of a patient’s expected death at home… discuss the use of EMS with the patient and/or family and inform them of the implication of using EMS (e.g., the involvement of EMS and risk of resuscitation attempts)” (Service 1, 2018) “When it has been determined that death has occurred, each paramedic will ensure that the Deceased Patient is treated with respect and dignity. It is important to be conscientious of the family and try to respect their wishes while concurrently ensuring that all appropriate notification(s) and detailed documentation requirements are completed in a timely manner.” (Service 7, 2019)
2. Generalized importance of family	6 (42.9%)	1 2 4 8 9 16	“Employees will continually strive towards being accountable in recognizing patient and families rights during the course of our contact with these individuals” (Service 4, 2018) “Paramedics will recognize and respectfully react to the needs of bystanders and patient family members, and will balance these needs with patient care demands” (Service 8, no date)
3. Family member escort options	8 (57.1%)	3 5 6 8 10 11 13 14	“Except in special circumstances, only one family member may accompany the patient. The family member must sit in the front passenger seat, with seatbelt fastened…” (Service 3, 2018) “The number of people travelling in an ambulance will not exceed the number of seatbelts available, and all passengers are required to use these according to Highway Traffic Act Regulations.” (Service 8, 2019) “Family Escorts of patients shall travel seated in the passenger seat following the vehicle safety restraints recommendations.” (Service 14, 2017)
4. Provider discretion	6 (42.9%)	5 6 10 11 12 14	“A family member (friend or co-worker) may accompany a patient being transported to hospital if in the judgement of the attending paramedic(s) that it will not compromise patient care or safety.” (Service 3, 2018) “It is understood that the attending paramedic has the final determination as to whether the family member rides in the patient’s or driver’s compartment.” (Service 6, 2016) “The limitations on a call will be a judgement decision made by the ambulance crew, at that time” (Service 11, 2016)
5. Family presence discouraged	3 (21.4%)	6 13 14	“Family and friends of the patient should be discouraged from accompanying patients in the ambulance, especially on Code 4 (Emergency) calls. Whenever possible, they should be encouraged to find and use alternate means of transportation.” (Service 13, 2007) “If under the discretion of the Paramedic crew the escort could cause more of a hindrance than assist in the patients care due to, but not limited to: Consideration for the potential of intense emotional reactions on the part of the family escort, as evidenced by the EMS personnel prior to transport. EMS personnel need to consider possible resuscitation or other critical interventions that may cause negative lasting effect on the Family Escort if witnessed during the transport” (Service 14, 2017)

### Experience-based co-design for family-centred prehospital cardiac arrest care

An output of our co-design workshops was our conceptualization of family-centred prehospital cardiac arrest care that incorporates content and themes identified in the qualitative document analysis, as well as survivor and family knowledge and experience, and family-centredness concepts [27–29]. See appendix figure three for our conceptual framework.

We also drafted a policy and procedure template to help improve the family-centredness of out-of-hospital cardiac arrest care. The template addresses six domains: doing what is best for the patient, family-centredness, assigning a family liaison, caring for the non-transported patient, providing written information and document best practices. The domains were constructed based on our qualitative analysis, meta-synthesis as well as team members knowledge and experience. See Table 3 for our policy and procedure template.

**Table 3:** Family-centred out-of-hospital cardiac arrest care policy and procedure template

Core content areas	Guiding principle(s)
Do what is best for the patient	Care of the patient in cardiac arrest is both the EMS provider and the family’s priority Patients should be stratified to hospitals best suited to caring for them, this may not always be the closest hospital (defer to local medical oversight) EMS organizations should employ evidence-based termination of resuscitation guidelines Families should make goals of care documents and advanced directives available for EMS providers as quickly as possible Disruptive persons, including family, should be removed from the scene if they interfere with resuscitation after limit setting and warning Removal of family members from the scene of resuscitation should not be the default directive of EMS organizations and should only be done when necessary Families acknowledge that EMS provider safety must take precedence over patient care
Family-centredness	When it is possible, and will not negatively impact the provision of care, EMS providers should acknowledge the family members on scene Inform the family of who you are and what you are there to do Whenever feasible, transport a family escort along with the patient Tell the family where the patient is being transported to and have a mechanism for updating them should the destination hospital change In circumstances where multiple family members are present, a single family representative should be identified EMS organizations will engage interdisciplinary committees that include patient and family representatives to develop, implement and evaluate policies and procedures
Assign a family liaison whenever possible	Once adequate providers are available to perform cardiac arrest care (airway management, medications, chest compressions, monitor/defibrillator, resuscitation team lead etc.), a family liaison should be assigned to the family and someone should be available as a contact person after the event The family liaison should offer and facilitate family presence during resuscitation, if the family wants it If it does not interfere with care, families should be allowed to touch, talk to, and/or pray for their family-member If the family does not want to be present during resuscitation, the family liaison should provide frequent updates and make reasonable accommodations for them that demonstrate caring i.e. therapeutic communication, assist with calling a support person etc If too few EMS providers are available to assign one the role of family liaison, the resuscitation team lead should perform this role as their workload allows If the clinical situation requires it, the family liaison can be reassigned to cardiac arrest care at the resuscitation team lead’s discretion
Caring for the non-transported	If the patient is not transported, and if it is feasible and not legally or operationally contraindicated, EMS providers should offer to move the deceased patient i.e. from floor to bed If the patient is not transported, EMS providers (ideally the family liaison) should take time to answer the family’s questions prior to leaving
Provide written information	Urgent bereavement services should be routinely engaged for families who have experienced the out-of-hospital cardiac arrest of a family member, especially if they were not transported EMS organizations should routinely provide written referral information for families experiencing critical incidents such as the cardiac arrest of a family member to critical incident stress debriefing and/or crisis counselling For the families of patients transported to hospital it is reasonable to defer responsibility for grief and bereavement services to hospital personnel Written information should be left for families who experience out-of-hospital cardiac arrest that includes: Who to contact with questions or concerns about the care provided Who to contact to request patient care records and information Who to contact to share gratitude and appreciation of the care provided Where to turn to get help for grief associated with experiencing cardiac arrest care
Important document features	Date policy and procedure developed Due date for next review Who to contact for suggestions revisions to policy and procedure Name of authors or committee responsible for policy and procedure development including patient and family representatives

## Discussion

### Interpretation

We collected and analyzed clinical governance documents related to family-centred care and cardiac arrest care in Canadian EMS organizations. Few documents were obtained that directed a family-centred approach. Organizational practices varied in their family-centredness from acknowledging the patient and family as a dyad to actively discouraging family involvement. No single organization’s documents included all components of family-centred cardiac arrest care identified in a recent review (i. primary focus on resuscitation; ii. consideration of family context, iii. collaboration between resuscitation team and family; iv. provisions for the family’s post-resuscitation needs; and iv. dedicated policies, procedures and education) [28], signalling a potential opportunity for improvement.

### Previous studies

Past research on prehospital family-centred care has identified numerous barriers to its provision including the belief it interferes with patient care, the unfamiliar environment, limited personnel, multi-tasking medical care and concern for patient care interference [13]. Adoption of family-centred care principles into training and clinical governance structures may help both front line EMS providers and physicians providing medical consultation overcome these barriers and improve care for their patients and families [27]. This is particularly relevant given that many EMS organizations are moving towards managing and terminating resuscitations on scene for reasons such as lack of benefit from transport, minimizing interruptions in CPR and minimizing exposure to additional health care providers [30, 31]. In these instances, undertaking simple low resource actions such as providing frequent updates, offering presence and answering questions may help families cope with the cardiac arrest experience.

### Strengths and limitations

This study analyzed clinical governance documents from Canadian EMS organizations that provide pre-hospital care to a large proportion of the population. Rather than just identifying a gap, this study contributes a potential solution by providing an experience-based co-designed policy and procedure template for future clinical governance document development.

Our results were obtained through a document review process which does not perfectly represent clinical care. Our request was for clinical governance documents related to family-centred care and cardiac arrest care; however, family-centredness may be built into other documents not obtained through our search. We also acknowledge that Canadian EMS organizations, specifically Canada’s highly trained and professional prehospital providers, are already providing family-centred care, regardless of the presence or absence of clinical governance documents. Furthermore, the EMS organizations that provided documents were primarily from provincial services and metropolitan centers, therefore EMS organizations from northern, Indigenous and French-speaking communities are under-represented in our findings. The impact of providing family-centred prehospital cardiac arrest care on scene times or important patient outcomes, is unknown.

### Clinical and research implications

Cardiac arrest of a family member is one of the most psychologically impactful and devastating life events [32–34]. Providing prehospital family-centred cardiac arrest care requires the ability to address intense emotional responses, address existential questions and competence in transitioning from patient care to family care [35]. To develop greater competence in family-centred care, and improve the experience of family members, organizational buy-in, training and clinical governance processes are required [22]. When successfully adopted, family-centredness is associated with higher quality of care and satisfaction for providers and families [36–38].

## Conclusion

We analyzed clinical governance documents related to family-centred care and cardiac arrest care from EMS organizations that care for approximately 80% of the population of Canada and found there were few directives to support family-centred care, especially as it relates to out-of-hospital cardiac arrest. Our qualitative document review employed patient and public involvement and identified various themes: addressing the family when there is a death in the home, the importance of family, family escort options and provider discretion. Informed by our document review, an experience-based co-design project was employed to create a family-centred out-of-hospital cardiac arrest care policy and procedure template to assist EMS organizations incorporate family-centredness in future clinical governance.

## Supplementary Information

Below is the link to the electronic supplementary material.

Supplementary file1 (DOCX 105 kb)

Supplementary file2 (DOCX 13 kb)

Supplementary file3 (DOCX 13 kb)

Supplementary file4 (DOCX 15 kb)

Supplementary file5 (DOCX 13 kb)

Supplementary file6 (DOCX 13 kb)

Supplementary file7 (DOCX 17 kb)

Supplementary file8 (DOCX 247 kb)
