# Supplementary material for: Caring for the invisible and forgotten: a qualitative document analysis and experience-based co-design project to improve the care of families experiencing out-of-hospital cardiac arrest
Source: CJEM. 2023 Feb 13;25(3):233–43. doi: 10.1007/s43678-023-00464-8 (PMC9924888; doi:10.1007/s43678-023-00464-8)
Supplement: Supplementary file 1 — Supplementary file1 (DOCX 105 kb) [file 43678_2023_464_MOESM1_ESM.docx]

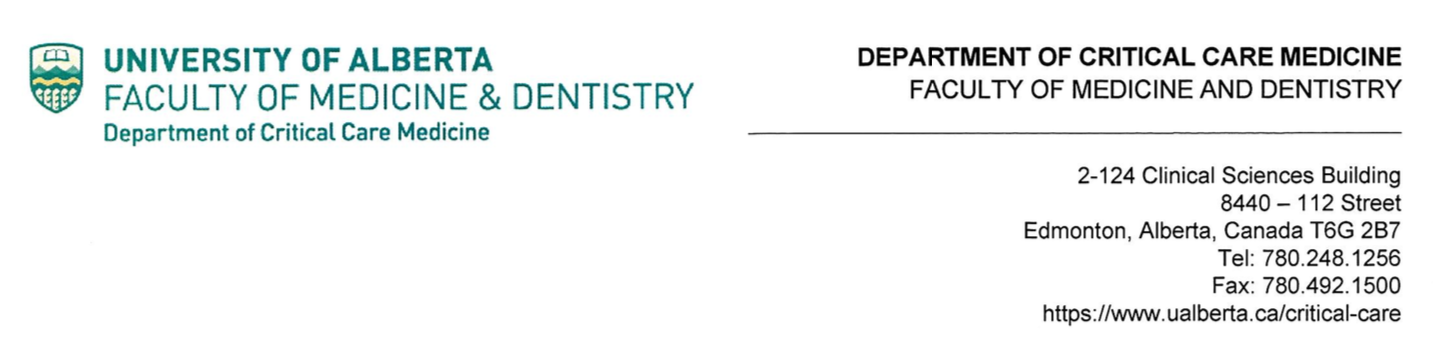
June 25^th^ 2020

Re: Family-centered care document review project

My name is [name and affiliation]. I am contacting you regarding a CanROC-related project about family-centered care in the pre-hospital setting. I am working with Matthew Douma, a CanROC researcher from the University of Alberta in Edmonton.

We are requesting **family-related policy and/or procedure documents** from CanROC participating sites like yours be emailed to [email address].

Any “clinical governance” type documents related to family presence, visitation and family transport, especially as they relate to cardiac arrest care are relevant. The documents will not be copied or shared and they will be anonymized, extracted, analyzed and deleted. Participating sites will receive a summary of our findings and a template family-centered care policy for prehospital care that is informed by this document review, a related systematic review of literature and an advisory committee of patients and families’ input.

This project has been granted an IRB bypass as it is a quality improvement project that does not involve humans or animal subjects. ARECCI Ethics Screening Tool. Link to bypass: <https://drive.google.com/file/d/1_mkwlNyCxVeCywxGyY2USqNNh1bP-a6k/view?usp=sharing>

Please scan and email family related documents to me at [email]. If you have any questions about this project, please contact Matthew.

Thank you for your time.

[name]


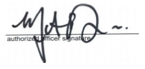


Matthew J Douma MN RN

Adjunct Associate Professor of Critical Care Medicine

(p) 780 233 9223 (e) douma@ualberta.ca
