# Supplementary material for: Caring for the invisible and forgotten: a qualitative document analysis and experience-based co-design project to improve the care of families experiencing out-of-hospital cardiac arrest
Source: CJEM. 2023 Feb 13;25(3):233–43. doi: 10.1007/s43678-023-00464-8 (PMC9924888; doi:10.1007/s43678-023-00464-8)
Supplement: Supplementary file 2 — Supplementary file2 (DOCX 13 kb) [file 43678_2023_464_MOESM2_ESM.docx]

**Table 4 (appendix). Family Centred Out of Hospital Cardiac Arrest Care Key Words**

| Family presence | Follow-up |
| --- | --- |
| Witnessed resuscitation | Debriefing |
| Patient centred/centered | Shared decision-making |
| Family centred/centered | Grief |
| Patient escort | Bereavement support |

Caption: these key words were used to assist in searching through documents obtained to determine their relevance to family centredness and cardiac arrest care.
