# Supplementary material for: Caring for the invisible and forgotten: a qualitative document analysis and experience-based co-design project to improve the care of families experiencing out-of-hospital cardiac arrest
Source: CJEM. 2023 Feb 13;25(3):233–43. doi: 10.1007/s43678-023-00464-8 (PMC9924888; doi:10.1007/s43678-023-00464-8)
Supplement: Supplementary file 3 — Supplementary file3 (DOCX 13 kb) [file 43678_2023_464_MOESM3_ESM.docx]

**Table 5.** **Internet Search Engine Strategy**

| Date searched: 12 to 16 October 2020 Results limited to Canada | | | | | |
| --- | --- | --- | --- | --- | --- |
| **Search** | **Search Terms** | **# results** | **# results screened** | **# new potentially relevant records** | **Total # records included** |
| 1 | “emergency medical services” AND “family centered” | ~ 131 000 | 200 | 0 | 0 |
| 2 | “emergency medical services” AND “patient and family centered” | ~ 65 100 | 200 | 0 | 0 |
| 3 | “emergency medical services” AND family | ~ 1 300 000 | 200 | 0 | 0 |
