# Supplementary material for: Caring for the invisible and forgotten: a qualitative document analysis and experience-based co-design project to improve the care of families experiencing out-of-hospital cardiac arrest
Source: CJEM. 2023 Feb 13;25(3):233–43. doi: 10.1007/s43678-023-00464-8 (PMC9924888; doi:10.1007/s43678-023-00464-8)
Supplement: Supplementary file 4 — Supplementary file4 (DOCX 15 kb) [file 43678_2023_464_MOESM4_ESM.docx]

**Table 6. Targeted Website Search Strategy and Results**

| Date searched: 12 to 16 October 2020  Search strings:  1) family centered care  2) patient and family centered care  3) family centred care  4) patient and family centred care | | | |
| --- | --- | --- | --- |
| **Website Searched** | **Link to relevant document(s)** | **Website Searched** | **Link to relevant document(s)** |
| www.paramedic.ca/ | 0 | [www.ambulancend.ca](http://www.ambulancend.ca) | 0 |
| www.[ahs.ca](http://sccm.org) | 0 | www.medavienb.ca | 0 |
| www.alberta.ca | 0 | [www.novascotia.ca](http://www.novascotia.ca) | 0 |
| www.bcehs.ca | 0 | [www.emci.ca](http://www.emci.ca) | 0 |
| www.gov.bc.ca | 0 | [www.ehsmfr.ca](http://www.ehsmfr.ca) | 0 |
| www.saskatchewan.ca | 0 | [www.princeedwardisland.ca](http://www.princeedwardisland.ca) | 0 |
| [www.athabascahealth.ca](http://www.athabascahealth.ca) | 0 | [www.islandems.ca](http://www.islandems.ca) | 0 |
| [www.gov.mb.ca](http://www.gov.mb.ca) | 0 | [www.gov.pe.ca](http://www.gov.pe.ca) | 0 |
| [www.sharedhealthmb.ca](http://www.sharedhealthmb.ca) | 0 | [www.gov.nl.ca](http://www.gov.nl.ca) | 0 |
| www.ontariohealth.ca | 0 | [www.westernhealth.nl.ca](http://www.westernhealth.nl.ca) | 0 |
| www.ontarioparamedic.ca | 0 | www.panl.ca | 0 |
| www.health.gov.on.ca | 0 | [www.yukon.ca](http://www.yukon.ca) | 0 |
| [www.oapc.ca](http://www.oapc.ca) | 0 | [www.yukonhositals.ca](http://www.yukonhositals.ca) | 0 |
| [www.lhsc.on.ca](http://www.lhsc.on.ca) | 0 | [www.hss.got.nt.ca](http://www.hss.got.nt.ca) | 0 |
| [www.countyofessex.ca](http://www.countyofessex.ca) | 0 | www.gov.nt.ca | 0 |
| [www.peelregion.ca](http://www.peelregion.ca) | 0 | www.gov.nu.ca | 0 |
| [www.memsochathamkent.com](http://www.memsochathamkent.com) | 0 | www.iqaluit.ca/ | 0 |
| www.mlems.ca | 0 | www.kawarthalakes.ca | 0 |
| [www.simcoe.a](http://www.simcoe.a) | 0 | [www.toronto.ca](http://www.toronto.ca) | 0 |
| www.niagraregion.ca | 0 | www.york.ca | 0 |
| [www.grey.ca](http://www.grey.ca) | 0 | [www.avenirensante.gouv.qc.ca](http://www.avenirensante.gouv.qc.ca) | 0 |
| www.memseo.com | 0 | [www.paramedic.quebec](http://www.paramedic.quebec) | 0 |
| [www.lambtononline.ca](http://www.lambtononline.ca) | 0 | www.emergensys.net | 0 |
| [www.haliburtoncountry.ca](http://www.haliburtoncountry.ca) | 0 | [www.urgences-sante.qc.ca](http://www.urgences-sante.qc.ca) | 0 |
| [www.corhealthontario.ca](http://www.corhealthontario.ca) | 0 | [www.quebec.ca](http://www.quebec.ca) | 0 |
| ww.kdsb.oc.ca | 0 | [www.2.gnb.ca](http://www.2.gnb.ca) | 0 |
| [www.halton.ca](http://www.halton.ca) | 0 | [www.horizonnb.ca](http://www.horizonnb.ca) | 0 |
| [www.leedsgrenville.com](http://www.leedsgrenville.com) | 0 | [www.perthcounty.ca](http://www.perthcounty.ca) | 0 |
| [www.northumberland.ca](http://www.northumberland.ca) | 0 | www.regionofwaterloo.ca | 0 |
