# Supplementary material for: Caring for the invisible and forgotten: a qualitative document analysis and experience-based co-design project to improve the care of families experiencing out-of-hospital cardiac arrest
Source: CJEM. 2023 Feb 13;25(3):233–43. doi: 10.1007/s43678-023-00464-8 (PMC9924888; doi:10.1007/s43678-023-00464-8)
Supplement: Supplementary file 5 — Supplementary file5 (DOCX 13 kb) [file 43678_2023_464_MOESM5_ESM.docx]

**Table 7. Grey Literature Database Search**

| **Date searched: 12 to 16 October 2020**  **Search Terms Used**  **1) Family AND EMS**  **2) Patient and Family Centered Care AND Prehospital/EMS/Ambulance** | |
| --- | --- |
| **Database** | **Relevant result** |
| [www.CMA.ca](http://www.cma.ca) | none |
| <http://www.ohri.ca/ksgroup/Publications.aspx> | none |
| <http://www.nlcahr.mun.ca/CHRSP/CompletedCHRSP.php> | none |
| <http://mchp-appserv.cpe.umanitoba.ca/deliverablesList.html> | none |
| <http://www.ihe.ca/index.php?/publications> | none |
| <http://www.inesss.qc.ca/en/publications/publications.html> | none |
| <http://hqca.ca/studies-and-reviews/completed-reviews/> | none |
| <https://acfp.ca/tools-resources/> | none |
| <https://www.alberta.ca/search-results.aspx?q=prone&p=1> | none |
| <https://www.hqontario.ca> | none |
| <https://www.sickkids.ca> | none |
| <https://muhc.ca> | none |
| <http://www.ohri.ca/home.asp> | none |
| <https://rnao.ca> | none |
| <https://wrha.mb.ca> | none |
| <https://www.tripdatabase.com> | none |
| <https://www.york.ac.uk/crd/> | none |
| <https://www.crd.york.ac.uk/PROSPERO/> | none |
