# Supplementary material for: Caring for the invisible and forgotten: a qualitative document analysis and experience-based co-design project to improve the care of families experiencing out-of-hospital cardiac arrest
Source: CJEM. 2023 Feb 13;25(3):233–43. doi: 10.1007/s43678-023-00464-8 (PMC9924888; doi:10.1007/s43678-023-00464-8)
Supplement: Supplementary file 6 — Supplementary file6 (DOCX 13 kb) [file 43678_2023_464_MOESM6_ESM.docx]

**Table 8. Patient and Public Involvement in the Supporting Family-Centred Cardiac Arrest Care Project GRIPP2 Short Form.**

| Aim | To perform a qualitative document analysis with survivor and family members aa co-investigators. To help improve the provision of patient and family centered cardiac arrest care in the prehospital setting. |
| --- | --- |
| Methods | Nineteen survivor and family partners were recruited from an existing Family Centred Cardiac Arrest Care online working group. They were involved in the conception of this project, refining the focus of the research questions, in developing the search strategy, in interpreting results and providing recommendations for future clinical governance document creation. Patient and family partners as co-investigators and collaborators contributed to writing and editing of this paper. |
| Results | Our co-investigators contributed to the project in several ways. Survivor and family co-investigators helped identify components of clinical governance documents that could impact the provision of patient and family centered care. Survivor and family co-investigators acted as subject matter experts for the researchers without lived experience to discuss and refine thematic analysis in addition to drafting the family centered cardiac arrest care template. Throughout the write-up phase for the patient and family partners contributed to manuscript preparation. |
| Discussion | Survivor and family co-investigator involvement in this project was effective and they made substantial contributions. The survivor and family co-investigators were involved from the beginning of the project allowing them to help shape the methods, analysis and subsequent recommendations. Pre-existing relationships with potential survivor and family co-investigators were an efficient and effective method for their recruitment to collaborate. However, there were limitations, having an online portal or forum could improve asynchronous work as the volume and frequency of emails was deemed to be too great for the PI to keep up with at times. |
| Reflections | Our survivor and family co-investigators occupied a position in our project that straddled traditional PPI and participatory research methods. PPI reporting methods such as the GRIPP2 were chosen as a best but imperfect fit. This work was unfunded and research support and coordination including administrative supports and technology support would have aided in the execution of this project. |
| GRIPP2  Source | http://wrap.warwick.ac.uk/91556/1/WRAP-GRIPP2-reporting-checklists-Staniszewska-2017.pdf |
