# Supplementary material for: Caring for the invisible and forgotten: a qualitative document analysis and experience-based co-design project to improve the care of families experiencing out-of-hospital cardiac arrest
Source: CJEM. 2023 Feb 13;25(3):233–43. doi: 10.1007/s43678-023-00464-8 (PMC9924888; doi:10.1007/s43678-023-00464-8)
Supplement: Supplementary file 8 — Supplementary file8 (DOCX 247 kb) [file 43678_2023_464_MOESM8_ESM.docx]

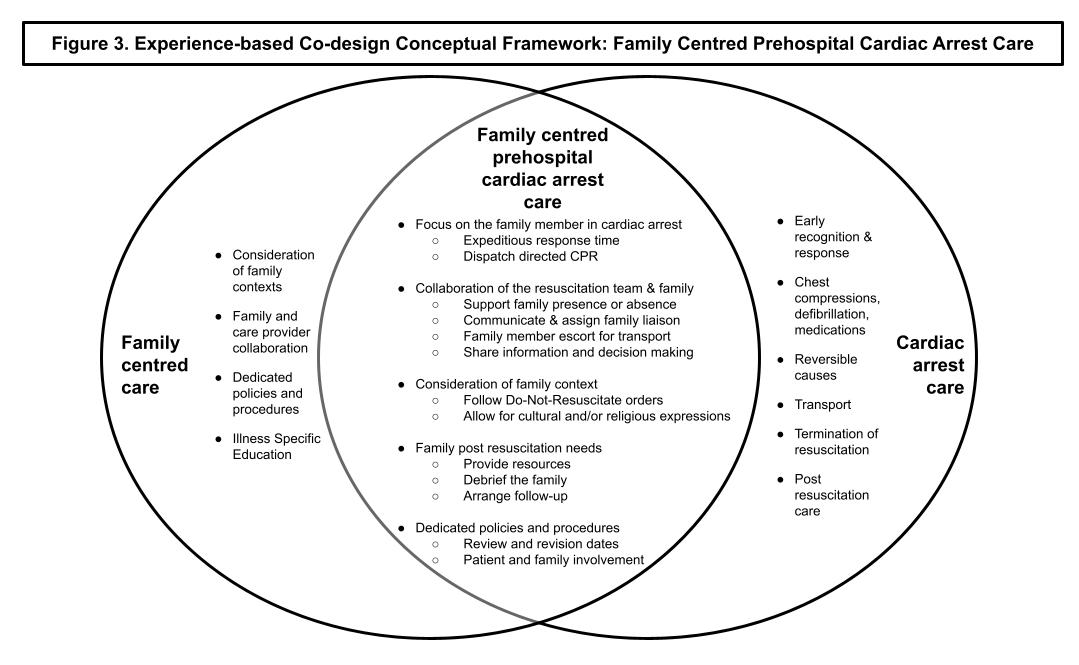


Caption: An output of our co-design workshops was our conceptualization of family centred prehospital cardiac arrest care (figure three) that incorporates content and themes identified in the qualitative document analysis, as well as survivor and family knowledge and experience, and family centredness concepts.
